# Using machine learning for healthcare treatment planning

**DOI:** 10.3389/frai.2023.1124182

**Published:** 2023-04-25

**Authors:** Snigdha Dubey, Gaurav Tiwari, Sneha Singh, Saveli Goldberg, Eugene Pinsky

**Affiliations:** ^1^Department of Computer Science, Metropolitan College, Boston University, Boston, MA, United States; ^2^Department of Radiation Oncology Mass General Hospital, Boston, MA, United States

**Keywords:** machine learning, ML in healthcare treatment, nearest neighbor classification, explainable AI, ML in healthcare environments

## Abstract

We present a methodology for using machine learning for planning treatments. As a case study, we apply the proposed methodology to Breast Cancer. Most of the application of Machine Learning to breast cancer has been on diagnosis and early detection. By contrast, our paper focuses on applying Machine Learning to suggest treatment plans for patients with different disease severity. While the need for surgery and even its type is often obvious to a patient, the need for chemotherapy and radiation therapy is not as obvious to the patient. With this in mind, the following treatment plans were considered in this study: chemotherapy, radiation, chemotherapy with radiation, and none of these options (only surgery). We use real data from more than 10,000 patients over 6 years that includes detailed cancer information, treatment plans, and survival statistics. Using this data set, we construct Machine Learning classifiers to suggest treatment plans. Our emphasis in this effort is not only on suggesting the treatment plan but on explaining and defending a particular treatment choice to the patient.

## 1. Introduction

Breast cancer is a leading cause of cancer-related deaths among women worldwide. Early detection and accurate breast cancer diagnosis are crucial for improving patient outcomes and reducing mortality rates. It is the most commonly diagnosed cancer type, accounting for 1 in 8 cancer diagnoses worldwide (CDC, 2022). According to the World Health Organization, in 2020, there were about 2.3 million new cases of breast cancer globally and about 685,000 deaths from this disease, with large geographical variations observed between countries and world regions (World health organization, 2022). Doctors often use additional tests to find or diagnose breast cancer. Breast cancer is treated in several ways. It depends on the kind of breast cancer and how far it has spread. People with breast cancer often get more than one kind of treatment.

1. Surgery
2. Chemotherapy
3. Hormonal therapy
4. Biological therapy
5. Radiation therapy

Machine learning (ML) algorithms have shown promise in aiding clinicians in the diagnosis, prognosis, and treatment of breast cancer. Among the different ML algorithms, logistic regression (LR), random forest (RF), and K-nearest neighbors (KNN) are widely used for breast cancer classification and prediction (e.g., Rajbharath and Sankari, 2017). These models have been shown to improve the accuracy and efficiency of breast cancer diagnosis, prognosis, and treatment planning.

Extensive literature (e.g., Ak, 2020) is present that compares the performance of multiple machine learning algorithms, including deep learning methods, in predicting breast cancer recurrence or classification. In our paper, the context is different. Our paper aims to provide a patient-centric approach to provide a dialogue between the physician and the patient. And keeping that as our focus, we concentrate on Machine Learning algorithms that are both explainable and accurate such as Logistic Regression.

The peculiarity of our study lies in the fact that the object of our approach is not a doctor who is offering the most optimal treatment method but a patient who is considering options for the treatment offered to him/her. This condition implies the following requirements and restrictions:

1. The need for dialogue between patient, doctor, AI.
2. The need to explain the AI decision.
3. Explanation of the decision should be in a form and terms understandable to the patient.

In this regard, an open database based on common and understandable symptoms and types of treatment was used. KNN was considered a system of explanation, the most simple and understandable form for the patient.

## 2. Methodology

In this paper, we suggest a methodology of using machine learning to help patients and doctors identify the appropriate treatment plan. For the case study, we have used breast cancer (Reddy et al., 2018; Song et al., 2021; van de Sande et al., 2021).

Our methodology for using Machine Learning consists of 2 stages:

**Stage 1:** In the first stage, we use state-of-the-art ML algorithms (Logistic Regression and Random Forest) (Bishop, 2016; Hastle, 2018) to find out the status of the patient after 5 years based on the Doctor's suggestion. We want to use a classifier that is sufficiently accurate and explainable. A good choice for such a classifier would be Logistic regression. On the other hand, there are classifiers, such as Random Forest, that often provide higher accuracy but are not explainable. Unless the accuracy of logistic regression is insufficient, this would be the classifier of choice. For our particular case study, the Random Forest classifier gives marginally better results, and therefore, logistic regression would be used.

**Stage 2:** In the second stage, we need to examine alternative treatment plans for a patient. To that end, we examine *k*-Nearest Neighbors (Cover and Hart, 1967; Sarkar and Leong, 2000; Bagui et al., 2003). We use statistics for these neighbors to examine alternative treatment plans such as:

1. Is chemotherapy required?
2. What is s the best radiation sequence with surgery?
3. What radiation recode should be proposed?

The idea is to help the doctor and the patient chooses a treatment to maximize the chances of survival after 5 years. We take *k* = 25 (neighbor) patients as we believe that this would be a number that is sufficient to compute statistics on alternative treatments and, at the same time, would allow the physician to examine these “neighbor” patients in detail and to explain the predicted results of alternative treatments.

We do not focus on *k*-nearest neighbors as an algorithm for breast cancer diagnosis, as considered in Medjahed et al. (2013). Our usage of nearest neighbors is to help the physician explain alternative treatments and outcomes once the prediction in Stage 1 is established. We should also note that the *k*-nearest neighbors require a distance metric, and one could get different results depending on the distance metrics and classification rules (Medjahed et al., 2013). In stage 2, we considered a distance metric where all features have the same weight. It is up to a physician to assign different weights depending on her/his expertise. However, it is easier for the patients to understand the similarity if the weights are the same. In general, to use KNN to explain the solution, one need's the concept of proximity from the user's point of view (Goldberg and Pinsky, 2022). In our case, the user is a patient, and the absence of symptoms signs in KNN may be necessary to start a dialogue with the doctor when explaining the proposed treatment.

This work also includes an interactive model where a patient can enter his details and use the model to predict the probability of his staying alive based on a combination of Radiation Sequence, Radiation Recode, and Chemotherapy Recode. For a survey of ML techniques in breast cancer prediction, see Boeri et al. (2020); Alaa et al. (2021), and Sugimoto1 et al. (2021).

## 3. The breast cancer dataset

For our research purpose, we requested access to Surveillance, Epidemiology, and End Results (SEER) custom breast cancer databases with the radiation and chemotherapy records' fields (SEER, 2022).

The data was accessed after signing the Data Use Agreement for SEER Radiation Therapy and Chemotherapy Information. Using the SEER*Stat tool, we selected the database named “November 2018 specialized databases,” which had additional treatment fields.

A Case listing session was created in order to fetch individual cancer records and patient histories. Fetching individual case listings allowed us to fetch the actual values stored in the database. We filtered the listings by specifying the cancer site to include only breast cancer cases.

We also segregated data based on the year of diagnosis and a selected number of intervals to be 5 years. We only included cases with a year of diagnosis > 2010 so that we have values for treatment-related fields.

The final step was to select the attributes/variables related to demographics, diagnosis, and treatment. The SEER*Stat query was executed, and the results were exported and saved as the Breast Cancer dataset.

The dataset can be accessed here: https://github.com/snehasi2703/BreastCancerSurvivalDataset.

**Figure 1:**
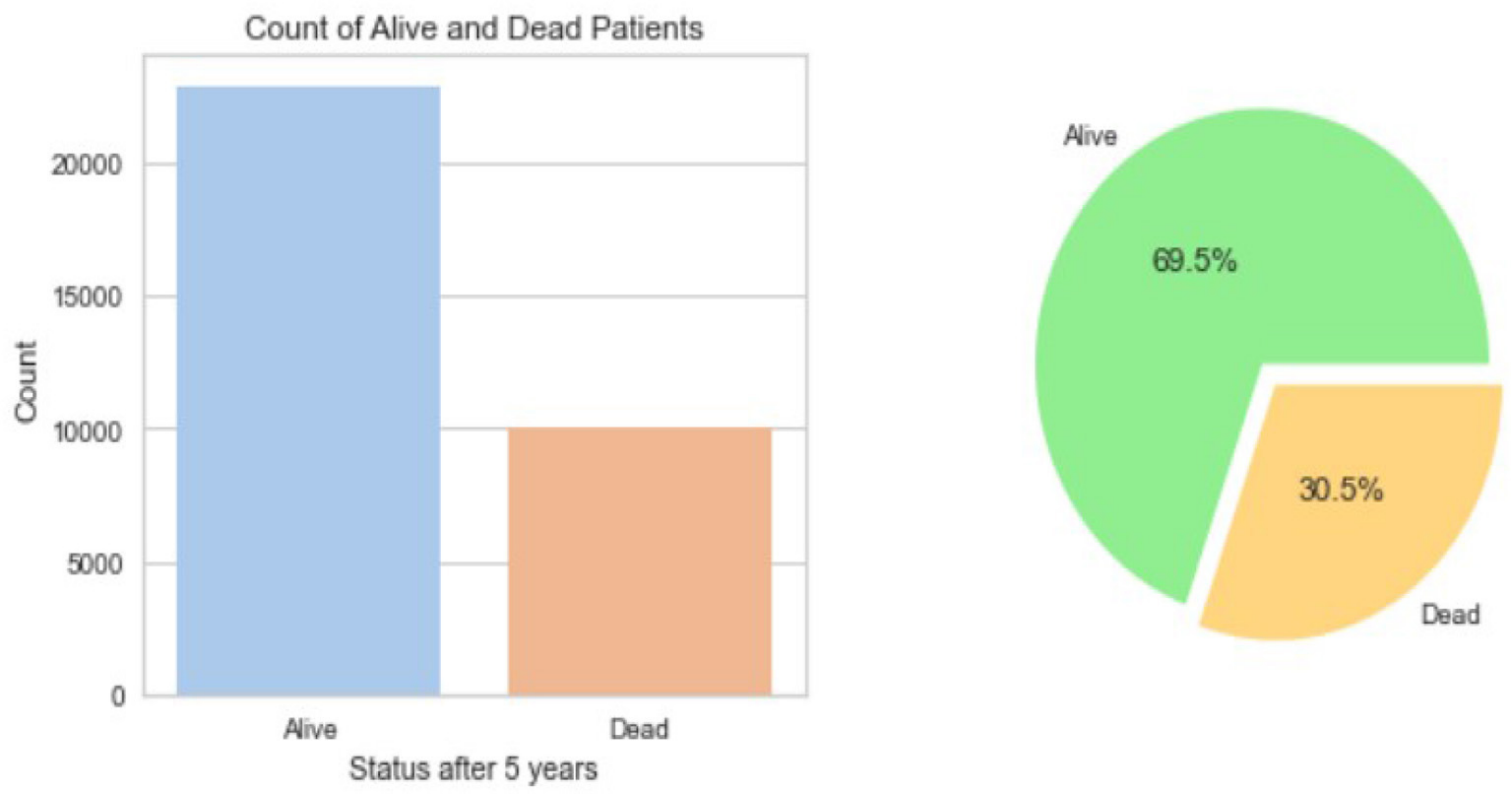
Dataset balance.

**Figure 2:**
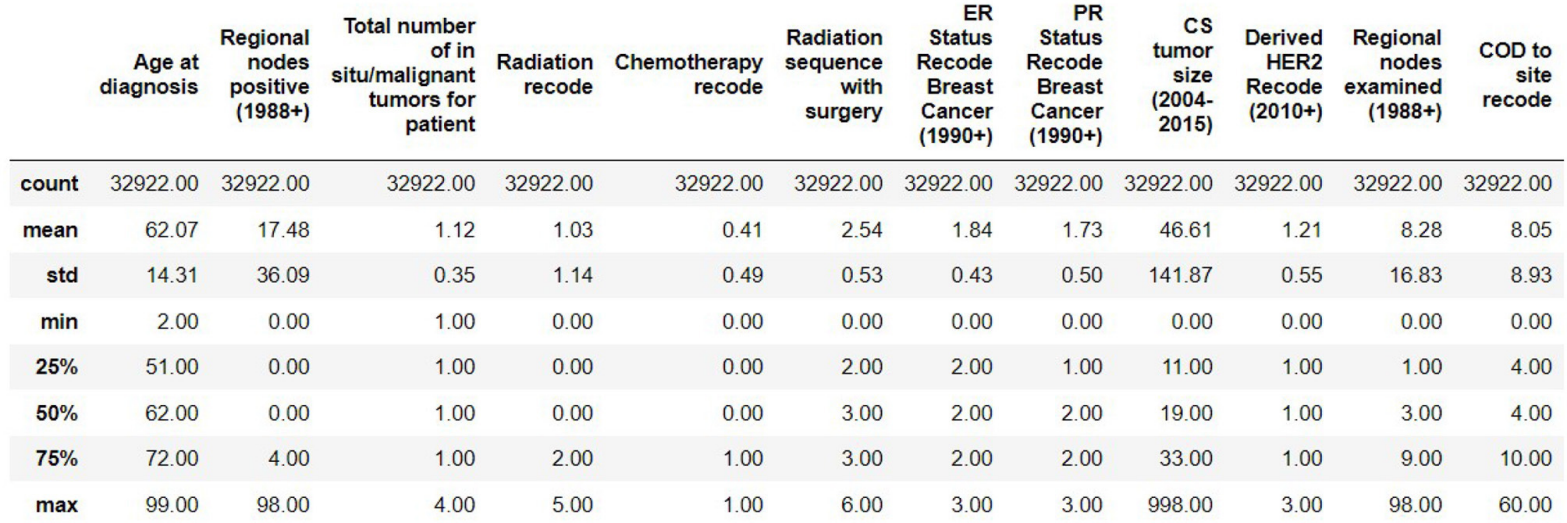
Dataset summary.

The resulting Breast Cancer dataset consists of 35,349 rows and 19 attributes (SEER, 2022). This Dataset is imbalanced. Out of 35,349 rows, 23,404 are alive patients at the end of 5 years, and 11,945 are as dead after different intervals (Figure 1). The dataset summary in Figure 2 shows the quartiles, medians, minimum, maximum, and means of all the required features considered at the time of Data cleaning.

### 3.1. Data cleaning

Out of the 35,349 records, there were 23,404 patients with Alive status while 11,945 with Dead status. The data set was then cleansed to remove all the NA values. The field CS Tumor Size had data with values 999, which meant that the tumor size for that patient's instance was unknown, or the size was not stated for that patient record. The data cleaning for such rows was also done to ensure only the valid instances where the tumor size is known are considered. Also, the field Regional Nodes Positive had a 99 in it, which meant that it is unknown whether the nodes are positive or not applicable and not stated in the patient record. Such data instances were also removed from the whole dataset to be used for model building. Post-data cleansing, the count of the entire dataset was 32,922 rows with 19 columns. Of these, 22,889 (69.5%) patient records had the status “Alive” while 10,033 (30.5%) had the status “Dead.” as shown in Figure 1. A detailed description of the types of the Features variables and their description is provided in Table 1.

**Table 1:** Dataset details.

**Attributes**	**Attributes description**	**Attributes type**
Age at diagnosis	Age at the beginning of treatment	Numeric
Regional nodes positive (1988+)	No. of regional lymph nodes positive, Above 90 unknown	Numeric
Total # of in situ/malignant tumors for patient	No. of malignant tumors for patient	Numeric
Radiation recode	Radiation type	Categorical
Chemotherapy recode	Chemotherapy done?	Categorical
Radiation sequence with surgery	Radiation sequence	Categorical
ER Status Recode Breast Cancer (1990+)	Estrogen receptor info	Categorical
PR Status Recode Breast Cancer (1990+)	Progesterone receptors info	Categorical
CS tumor size (2004-2015)	Tumor size	Numeric
Derived HER2 Recode (2010+)	Joint hormone receptor	Categorical
Regional nodes examined (1988+)	Records the total # of regional lymph nodes that were removed	Numeric
COD to site recode	Cause of Death	Categorical
Race Recode	Race categories	Categorical
Sex	Sex of patient	Categorical
Vital status recode (study cutoff used)	Patient Status	Categorical
Diagnosis_year	Year when diagnosis started	Numerical
Last_fu _year	Last Year of contact for treatment	Numerical
Interval_years	Number of Intervals the screening was done	Numerical
Status_5_years	Status of the patient during 5 years	Categorical

### 3.2. Feature selection

**Figure 3:**
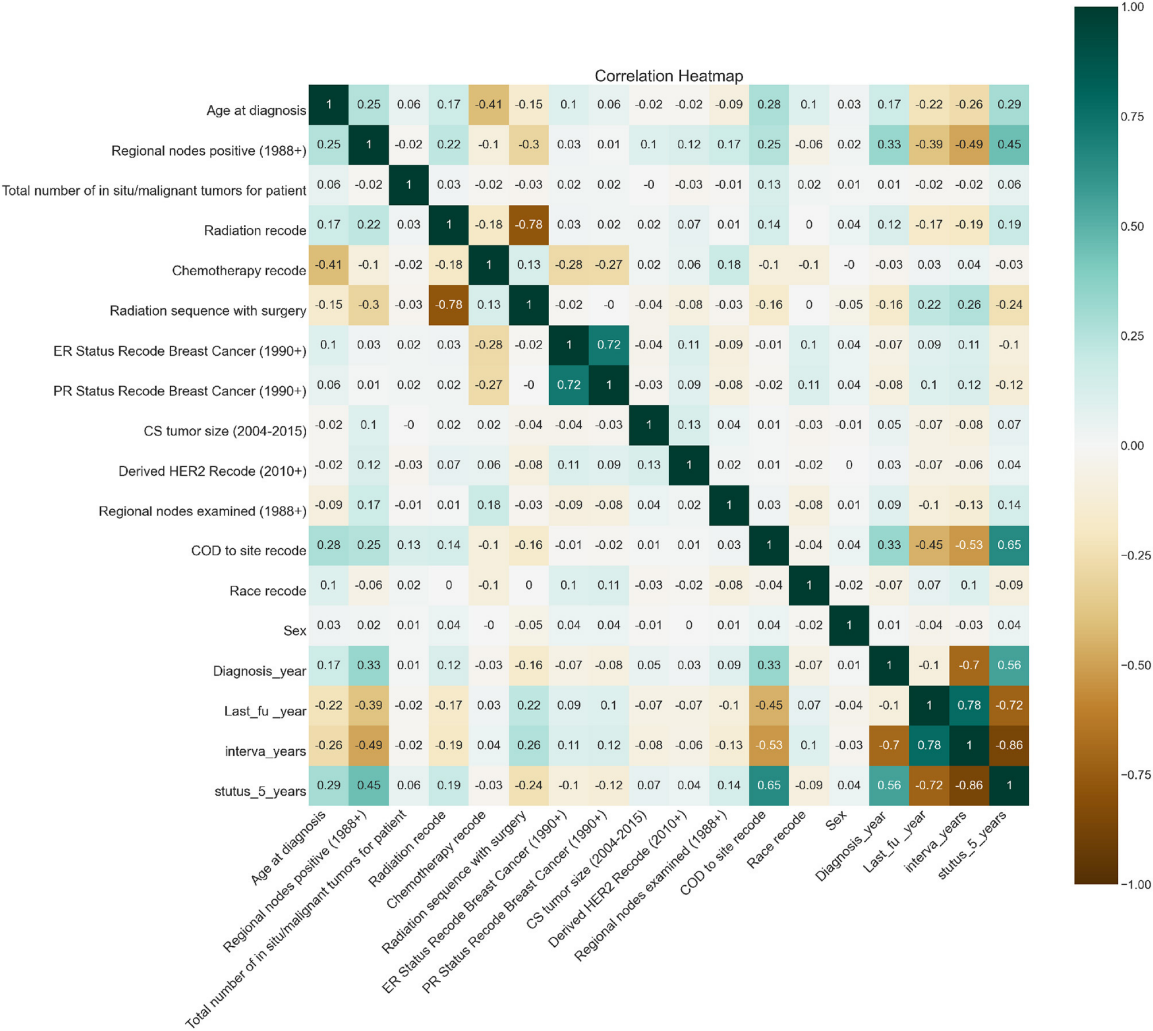
Correlation heatmap.

**Figure 4:**
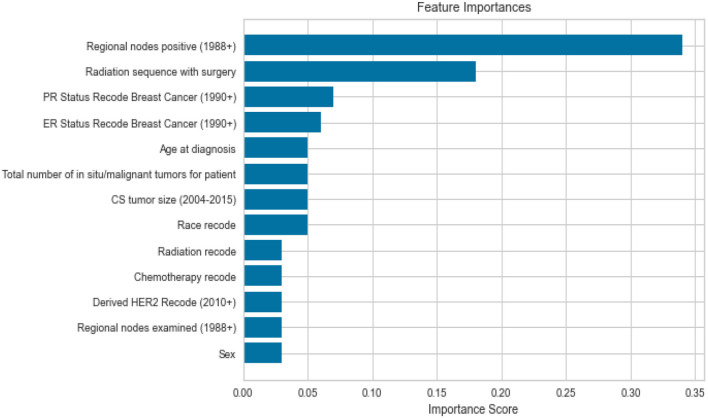
Feature importance.

Feature importance was also taken into account to avoid missing important features. Out of 19 features, we removed all the features which were highly correlated with the class label as shown in Figure 3 since they were contributing 96 percent to the performance of the model, which was earlier 99 percent. The correlation was taken into consideration to improve the reliability of the model. Highly correlated attributes with the Target Variable contributed to the increased accuracy as they were directly associated with the Target Variable. Correlation details of the features with the target variable are shown in detail in Table 2. Feature importance and their scores are shown in Figure 4.

**Table 2:** Attribute importance and correlation with target variable.

**Attribute**	**Importance**	**Correlation with target variable**
Regional nodes positive (1988+)	0.34	0.45
Radiation sequence with surgery	0.18	–0.24
PR Status Recode Breast Cancer (1990+)	0.07	–0.12
ER Status Recode Breast Cancer (1990+)	0.06	–0.10
Age at diagnosis	0.05	0.29
Total number of in situ/malignant tumors for patient	0.05	0.06
CS tumor size (2004-2015)	0.05	0.07
Race recode	0.05	–0.09
Radiation recode	0.03	0.19
Chemotherapy recode	0.03	–0.03
Derived HER2 Recode (2010+)	0.03	0.04
Regional nodes examined (1988+)	0.03	0.14
Sex	0.03	0.04
COD to site recode	**	0.65
Diagnosis_year	**	0.56
Last_fu _year	**	–0.72
Interva_years	**	–0.86

^**^Feature not considered for study due to high correlation with Target Variable.

## 4. ML models and performance evaluation

We chose Logistic Regression as the model for its easier explainability and the fact that it doesn't require any hyperparameter tuning and Random Forest to support our idea that Logistic Regression acts as a good classifier and results are on par with Random Forest. KNN was used for the explanation on the basis of past nearest patients having similar characteristics and to answer the questions of the patients and look at the previously evaluated patients and what worked in their cases.

### 4.1. Logistic regression

Logistic regression is a statistical method used in machine learning to predict the probability of an outcome being one of two possible classes, given one or more independent variables. It is a binary classification algorithm that estimates the relationship between the independent variables and the dependent variable, using a logistic function to transform the output into a probability value.

### 4.2. Random forest

Random Forest is a machine learning algorithm used for both classification and regression problems. It is an ensemble learning method that combines multiple decision trees, each trained on a subset of the available features and data. Random Forest randomly selects features and data samples for each decision tree and aggregates the output of all the trees to make a final prediction.

The individual decision trees in a Random Forest model are trained using a technique called bagging (bootstrap aggregating), which involves resampling the data with replacement to create multiple training sets. The output of each decision tree is combined to produce a more accurate and stable prediction than a single decision tree.

The default parameters were used to initialize the model, and then we tuned the parameters to adjust the model to achieve the best performance, and we settled with max_depth as 10.

### 4.3. K nearest neighbors

K Nearest Neighbors (KNN) is a simple machine-learning algorithm for classification and regression problems. It predicts the value of an input data point based on the most frequent class or average value of the k nearest neighbors in the training set. KNN is often used for small data sets with complex relationships between input and output variables. However, it can be computationally expensive and sensitive to the choice of distance metric and the number of neighbors *k*.

We took the 25 nearest neighbors because this number is neither low nor high enough for a the physician to manually go ahead and verify by looking at 25 patients close to the patient.

### 4.4. Model training and performance evaluation

There were a total of 13 features (after removing COD to Site Recode, Diagnosis_year, Last_fu_year, interva_years, and Vital Status Recode) that we used to build our ML model for predicting the status of the patient after 5 years. A detailed description of these features is given in Table 1. We encoded these features using Label Encoder for the categorical values and then split the data into 50/50 with stratified data sampling across stutus_5_years and random_state as 42.

We built two prediction models: one using a Random Forest Classifier and another using Logistic regression. Both models utilized these 13 features to predict the patient's status after the number of intervals. The performance of the models was computed using the Confusion Matrix, Area Under the Curve (AUC), and *F*_1_ Score. We chose the AUC and *F*_1_ Score to understand how the model performs with each label broadly.

AUC is a measure of the performance of a classification model that quantifies how well the model can distinguish between positive and negative classes. The AUC represents the area under the receiver operating characteristic (ROC) curve, which plots the true positive rate (TPR) against the false positive rate (FPR) at different classification thresholds (Bishop, 2016). Its formula is given by


$$\begin{array}{l} \begin{array}{l} \text{AUC}=\int_{1}^{0}\text{TPR}(\text{FP}{\text{R}}^{-1}(t))dt \end{array} \end{array}$$

The AUC ranges from 0 to 1, with a higher AUC indicating a better performance of the model. An AUC of 0.5 indicates that the model performs no better than random guessing, while an AUC of 1 indicates perfect classification.

By contrast, the *f*_1_-score is a measure of the balance between the precision and recall of a classification model. It is the harmonic mean of precision and recall, with a value between 0 and 1, where a higher value indicates better performance. *f*_1_-score is particularly useful when the data is imbalanced. Formula:


$$\begin{array}{l} F1=2\cdot \frac{\text{precision }\times \text{ recall}}{\text{precision }+\text{ recall}} \end{array}$$

where:


$$\begin{array}{l} \text{precision}=\frac{TP}{TP+FP}\;and\text{ recall}=\frac{TP}{TP+FN} \end{array}$$

A confusion matrix is a table used to evaluate the performance of a classification model by comparing the actual and predicted classes of a set of data. The table has four entries: true positives (TP), false positives (FP), true negatives (TN), and false negatives (FN).

The detailed results are shown in the results section. We also wanted to understand how the spread of data points happens across different age groups and how will the model performance change or gets impacted for different age groups of patient records. For that, we split the data into three ranges of age: 0 − 45, 45 − 65, and age >65 years.

We also looked at the cross-validation score of the models to verify the robustness of the predictive model and their performance across the different age groups.

## 5. Results and discussion

Tables 3, **5** present the performance metrics of the model for the full dataset, as well as for different age groups.

**Table 3:** Logistic regression model results.

**Specifications**	**True positives**	**True negatives**	**False positives**	**False negatives**	**Recall**	**Specificity**	**FPR**	**FNR**	**Precision**	**Accuracy**	**AUC**	**F1 Score**
Full dataset	10,658	2,375	787	2,641	0.80	0.75	0.25	0.20	0.93	0.79	0.70	0.58
0-45 Years age	1,573	113	82	323	0.83	0.58	0.42	0.17	0.95	0.81	0.60	0.36
45-65 Years age	5,886	622	250	1,065	0.85	0.71	0.29	0.15	0.96	0.83	0.66	0.49
Age > 65 years	3,062	1,792	592	1,102	0.74	0.75	0.25	0.26	0.84	0.74	0.73	0.68

The specifications column lists the different subsets of the data based on age, while the remaining columns present the following performance metrics:

- **True Positives:** The number of individuals who were correctly identified as having the condition.
- **True Negatives:** The number of individuals who were correctly identified as not having the condition.
- **False Positives:** The number of individuals who were incorrectly identified as having the condition (also known as Type I error).
- **False Negatives:** The number of individuals who were incorrectly identified as not having the condition (also known as Type II error).
- **Recall:** The proportion of true positives out of all actual positives. This metric measures how well the model identifies individuals with the condition.
- **Specificity:** The proportion of true negatives out of all actual negatives. This metric measures how well the model identifies individuals without the condition.
- **FPR (False Positive Rate):** The proportion of false positives out of all actual negatives. This is the complement of specificity.
- **FNR (False Negative Rate):** The proportion of false negatives out of all actual positives. This is the complement of the recall.
- **Precision:** The proportion of true positives out of all predicted positives. This metric measures how many of the predicted positives are actually true positives.
- **Accuracy:** The proportion of correctly classified individuals out of all individuals.
- **AUC (Area Under the Curve):** The area under the ROC curve measures the trade-off between recall and specificity for different classification thresholds. A higher AUC indicates better classification performance.
- **F1 Score:** The harmonic mean of precision and recall. This metric provides a balanced measure of the model's ability to identify both true positives and true negatives.

### 5.1. Logistic regression performance

**Figure 5:**
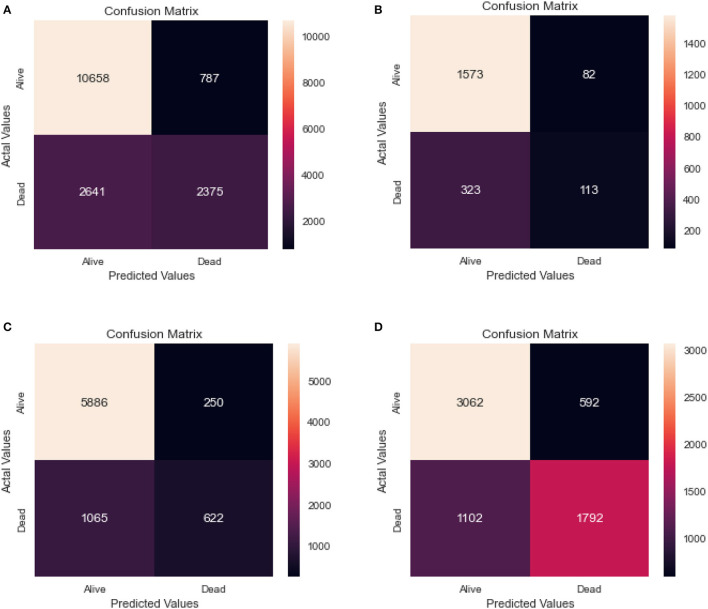
Confusion matrix of logistic regression. **(A)** Full dataset using LR. **(B)** Dataset of age < 45 years using LR. **(C)** Dataset of 45<age<65 years using LR. **(D)** Dataset of age > 65 years using LR.

Table 3 shows the results of a logistic regression model that was used to classify individuals as either alive or dead. The confusion matrix for the same model for the different age groups is shown in Figure 5.

From the results, we can see that the full dataset achieved an accuracy of 0.79, with a relatively high AUC of 0.70. The recall and specificity were 0.80 and 0.75, respectively, indicating a reasonable balance between identifying true positives and true negatives. The precision was high at 0.93, indicating that a large majority of the predicted positives were actually true positives.

The table also shows the results for different age groups. We can see that the model performed relatively well for all age groups, with the highest performance in the 45–65 age group. This group had the highest recall and specificity, as well as the highest AUC and F1 score. The age group above 65 years had the lowest recall, while the 0–45 age group had the lowest precision and F1 score.

10-fold cross-validation is a common training and validating method. It randomly divides the dataset into ten subsets, each turn of a total of ten in the validation process chooses one subset as the testing dataset, and the remaining nine are the training dataset. The average value of the accuracy (or error rate) of the ten times the results were used as the estimation of the accuracy of the algorithm.

We also cross-validated our models for the model robustness, and we obtained the below results for the different age groups as shown in Table 4.

**Table 4:** Logistic regression model cross validation results.

**Specifications**	**Cross-val score**	**Average accuracy**
Full dataset	0.78, 0.78, 0.76, 0.80, 0.79, 0.80, 0.78, 0.78, 0.79, 0.79	0.78
0-45 Years age	0.82, 0.83, 0.83, 0.81, 0.81, 0.81, 0.81, 0.79, 0.81, 0.80	0.81
45-65 Years age	0.84, 0.83, 0.83, 0.83, 0.82, 0.83, 0.83, 0.82, 0.84, 0.83	0.83
Age > 65 years	0.77, 0.73, 0.74, 0.73, 0.75, 0.74, 0.75, 0.72, 0.74, 0.77	0.74

### 5.2. Random forest performance

**Figure 6:**
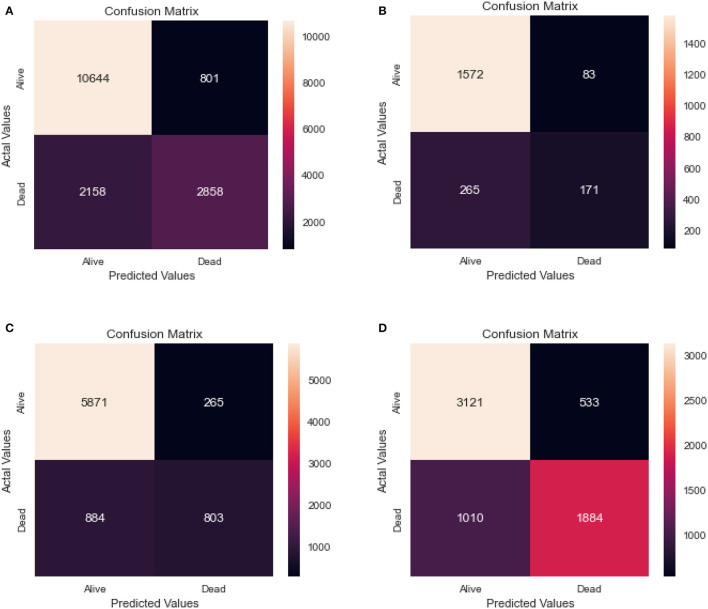
Confusion matrix of random forest. **(A)** Full dataset using RF. **(B)** Dataset of age < 45 years using RF. **(C)** Dataset of 45<age<65 years using RF. **(D)** Dataset of age > 65 years using RF.

Table 5 shows the results of a Random Forest model that was used to classify individuals as either alive or dead. The confusion matrix for the same model for the different age groups is shown in Figure 6.

**Table 5:** Random forest model results.

**Specifications**	**True positives**	**True negatives**	**False positives**	**False negatives**	**Recall**	**Specificity**	**FPR**	**FNR**	**Precision**	**Accuracy**	**AUC**	**F1 Score**
Full dataset	10,644	2,858	801	2,158	0.83	0.78	0.22	0.17	0.93	0.82	0.75	0.66
0-45 Years age	1,572	171	83	265	0.86	0.67	0.33	0.14	0.95	0.83	0.67	0.50
45-65 Years age	5,871	803	265	884	0.87	0.75	0.25	0.13	0.96	0.85	0.72	0.58
Age > 65 years	3,121	1,884	533	1,010	0.76	0.78	0.22	0.24	0.85	0.76	0.75	0.71

The full dataset has a recall of 0.83, specificity of 0.78, FPR of 0.22, FNR of 0.17, precision of 0.93, the accuracy of 0.82, AUC of 0.75, and F1 score of 0.66. The 0–45 years age group has a recall of 0.86, specificity of 0.67, FPR of 0.33, FNR of 0.14, the precision of 0.95, accuracy of 0.83, AUC of 0.67, and F1 score of 0.50. The 45–65 years age group has a recall of 0.87, specificity of 0.75, FPR of 0.25, FNR of 0.13, the precision of 0.96, accuracy of 0.85, AUC of 0.72, and F1 score of 0.58. Finally, the age over 65 years group has a recall of 0.76, specificity of 0.78, FPR of 0.22, FNR of 0.24, the precision of 0.85, accuracy of 0.76, AUC of 0.75, and F1 score of 0.71.

The results suggest that the model performs well overall, with high recall and precision scores. However, it appears that the model performs slightly better for the age 45-65 years group, with higher scores for most performance metrics compared to the other age groups.

We also cross-validated our models for the model robustness, and we obtained the below results for the different age groups as shown in Table 6.

**Table 6:** Random forest model cross validation results.

**Specifications**	**Cross-val score**	**Average accuracy**
Full dataset	0.82, 0.80, 0.79, 0.82, 0.83, 0.82, 0.82, 0.81, 0.81, 0.82	0.81
0–45 Years age	0.86, 0.87, 0.87, 0.81, 0.81, 0.87, 0.83, 0.82, 0.80, 0.84	0.85
45–65 Years age	0.87, 0.85, 0.85, 0.85, 0.85, 0.86, 0.86, 0.86, 0.86, 0.85	0.86
Age > 65 years	0.77, 0.76, 0.75, 0.76, 0.77, 0.77, 0.77, 0.77, 0.77, 0.77	0.77

### 5.3. Comparing both models

**Figure 7:**
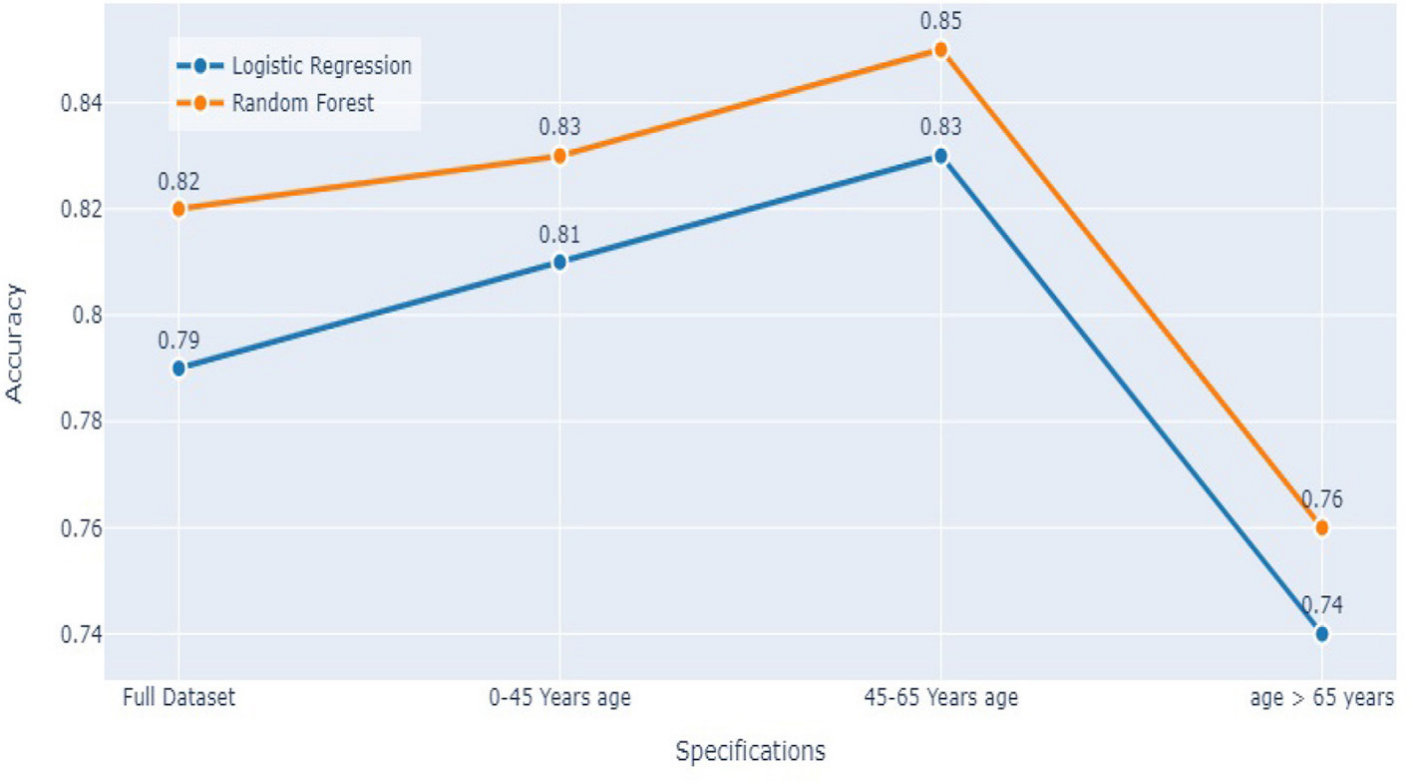
Models accuracy comparison.

**Figure 8:**
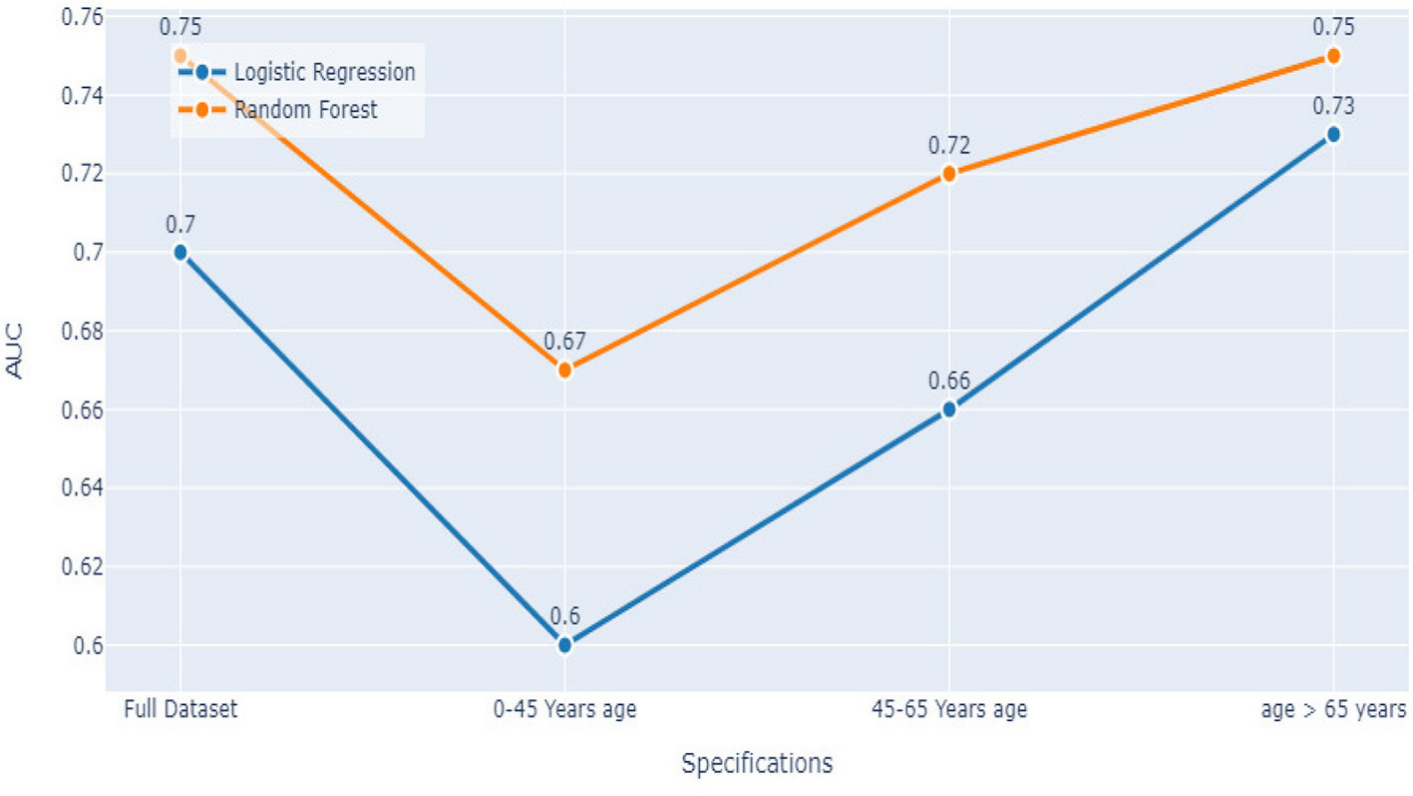
Models AUC score comparison.

**Figure 9:**
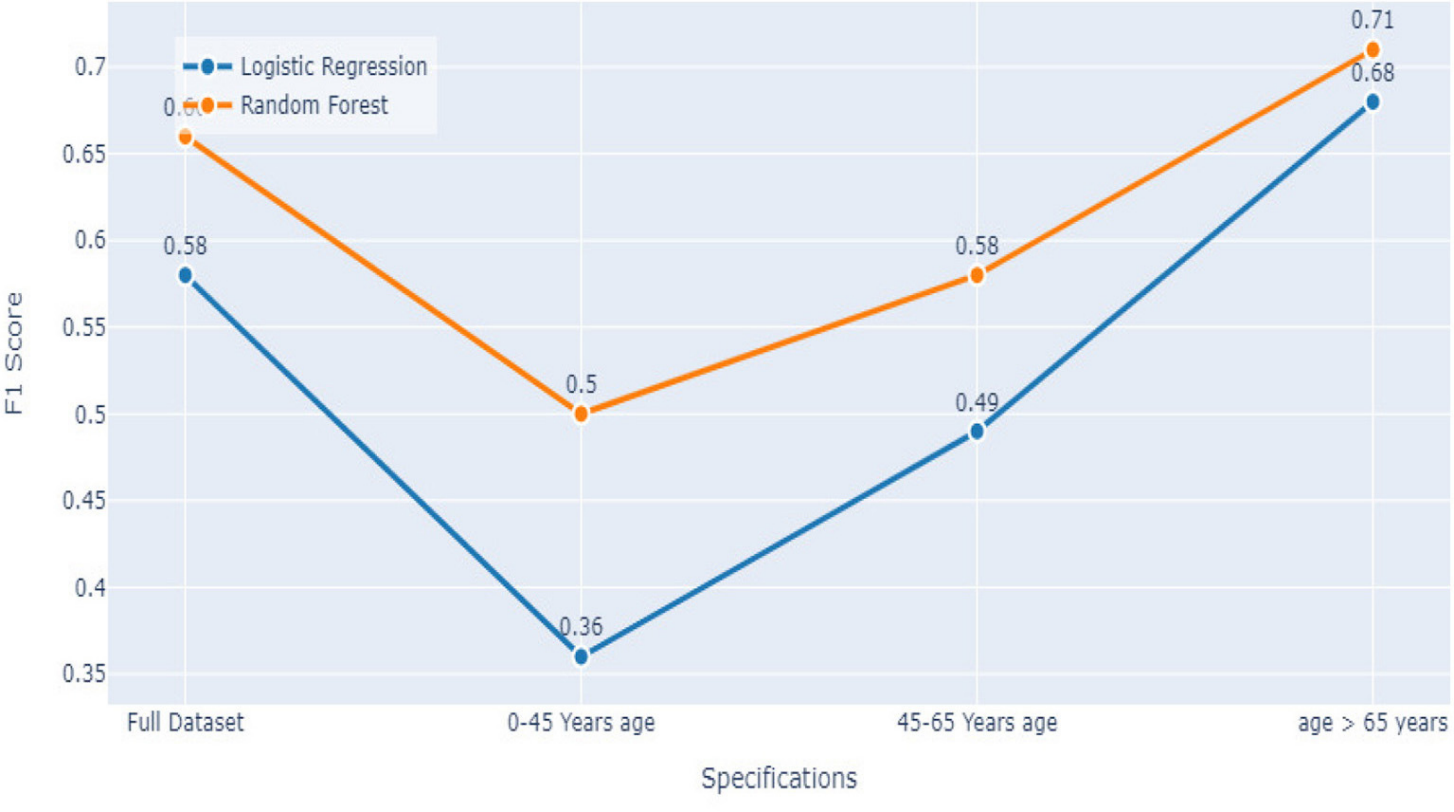
Models F1 score comparison.

Comparing the results of the logistic regression in Table 3 with the results of the Random Forest classifier in Table 5, we notice that the Logistic regression gives competitive results. Models Accuracy for the different age groups for the Logistic and Random Forest model is shown in Figure 7. Comparison of AUC Scores of the different age groups for the Logistic and Random Forest model is shown in Figure 8. While comparison of F1 Scores of the different age groups for the Logistic and Random Forest model is shown in Figure 9. These figures clearly illustrate that the performance of the Logistic Regression model is at par with Random Forest Model. The True Positives of Logistic regression on the Full dataset are higher than Random Forest, 10,658 and 10,644, respectively. Unlike the Random Forest classifier, the logistic regression is explainable and allows us to rank the importance of features. Even the results of the Logistic Regression models can be replicated again on multiple runs, unlike Random Forest, whose prediction could change on simultaneous runs, which makes us hesitant to use Random Forest for medical cases.

Therefore, we decided to use the logistic regression for Stage 1 of our analysis.

Once the patient status is predicted, we consider the second stage: we implemented *k*-NN to find 25 nearest neighbors to the patient and advise the patient regarding Chemotherapy and Radiation sequence treatments. For this, we have included only the “Alive” patient Dataset to increase the chances of survival for the patient. We took 25 nearest neighbors because this number is neither low nor high enough for a physician to manually go ahead and verify by looking at 25 patients close to the patient in question.

To illustrate our approach, we consider two patients, Patient_1 and Patient_2. Their features are summarized in Table 7.

**Table 7:** Sample data for two patients.

**Feature**	**Patient_1**	**Patient_2**
Age at diagnosis	67	57
Regional nodes positive (1988+)	2	0
Total # of in situ/malignant tumors	1	1
Radiation recode	0	0
Chemotherapy recode	1	1
Radiation Sequence with Surgery	3	3
ER Status Recode Breast Cancer (1990+)	2	1
PR Status Recode Breast Cancer (1990+)	2	1
CS tumor size (2004–2015)	70	11
Derived HER2 Recode (2010+)	1	2
Regional nodes examined (1988+)	21	1
Race recode	0	2
Sex	0	0
Interval years	5	5

We used Logistic Regression for the prediction of the survival of the patient after 5 years if the patient follows the advice of the doctor and also the recommendations of the doctor. We are also predicting the survival chances if the patient decides not to undergo radiation and chemotherapy. Then using K Nearest Neighbors, we check the probability of the survival of the patient for various combinations of Chemotherapy and Type of Radiation and Radiation Sequence.

We start with the first patient, Patient_1. According to our model, the doctor has advised the patient to Beam Radiation and chemotherapy. Based on the Logistic Regression patient would be alive after 5 years if the patient follows the doctor's advice or even if the patient refuses the doctor's advice.

*k*-Nearest Neighbors of 25 resulted in the probability of the patient being alive after 5 years to 68%. But if the patient refuses to take the Beam radiation, the model predicts the probability of the patient being alive after 5 years as 0%. In case the the patient refuses any radiation and also wants no chemotherapy too, then the probability of the patient being alive after 5 years reaches 4%.

Now, consider the second patient, Patient_2. For this specific patient, according to our model, the doctor has advised the patient to Beam Radiation and also to take chemotherapy. Based on the Logistic Regression patient would be dead after 5 years if the patient follows the doctor's advice or even if the patient refuses the doctor's advice. *K* Nearest Neighbors of 25 resulted in the probability of the patient being alive after 5 years to 84%. But if the patient refuses to take the Chemotherapy, the model can predict the probability of the patient being alive after 5 years as 76%. In case the patient refuses any radiation and also wants no chemotherapy too, then the probability of the patient being alive after 5 years reaches 4%.

Using *k* = 25 Nearest Neighbors, patients and doctors can examine several combinations of “Chemotherapy Recode,” “Radiation Recode,” and “Radiation Sequence” and have the probabilities of survival after 5 years using the interactive model. This would allow them to decide on the more appropriate treatment as shown in Tables 8, 9.

**Table 8:** Scenarios for Patient_1.

**Scenario for patient 1**	**Radiation recode**	**Chemotherapy recode**	**Radiation sequence with surgery**	**Probability**
Scenario 1	Beam Radiation	Yes	Radiation after Surgery	68
Scenario 2	Beam Radiation	No/Unknown	Intraoperative rad with other rad before/after surgery	20
Scenario 3	Beam Radiation	No/Unknown	Intraoperative radiation	56
Scenario 4	Beam Radiation	No/Unknown	Radiation before and after surgery	8
Scenario 5	Beam Radiation	No/Unknown	Radiation prior to surgery	40
Scenario 6	Refused	No/Unknown	Radiation after surgery	4

**Table 9:** Scenarios for Patient_2.

**Scenario for patient 2**	**Radiation recode**	**Chemotherapy recode**	**Radiation sequence with surgery**	**Probability**
Scenario 1	Beam Radiation	Yes	Radiation after Surgery	84
Scenario 2	Beam Radiation	No/Unknown	Radiation after Surgery	76
Scenario 3	Beam Radiation	No/Unknown	Intraoperative rad with other rad before/after surgery	20
Scenario 4	Beam Radiation	No/Unknown	Intraoperative radiation	56
Scenario 5	Beam Radiation	No/Unknown	Radiation before and after surgery	8
Scenario 6	Beam Radiation	No/Unknown	Radiation prior to surgery	40
Scenario 7	Refused	No/Unknown	Radiation after surgery	4

**Figure 10:**
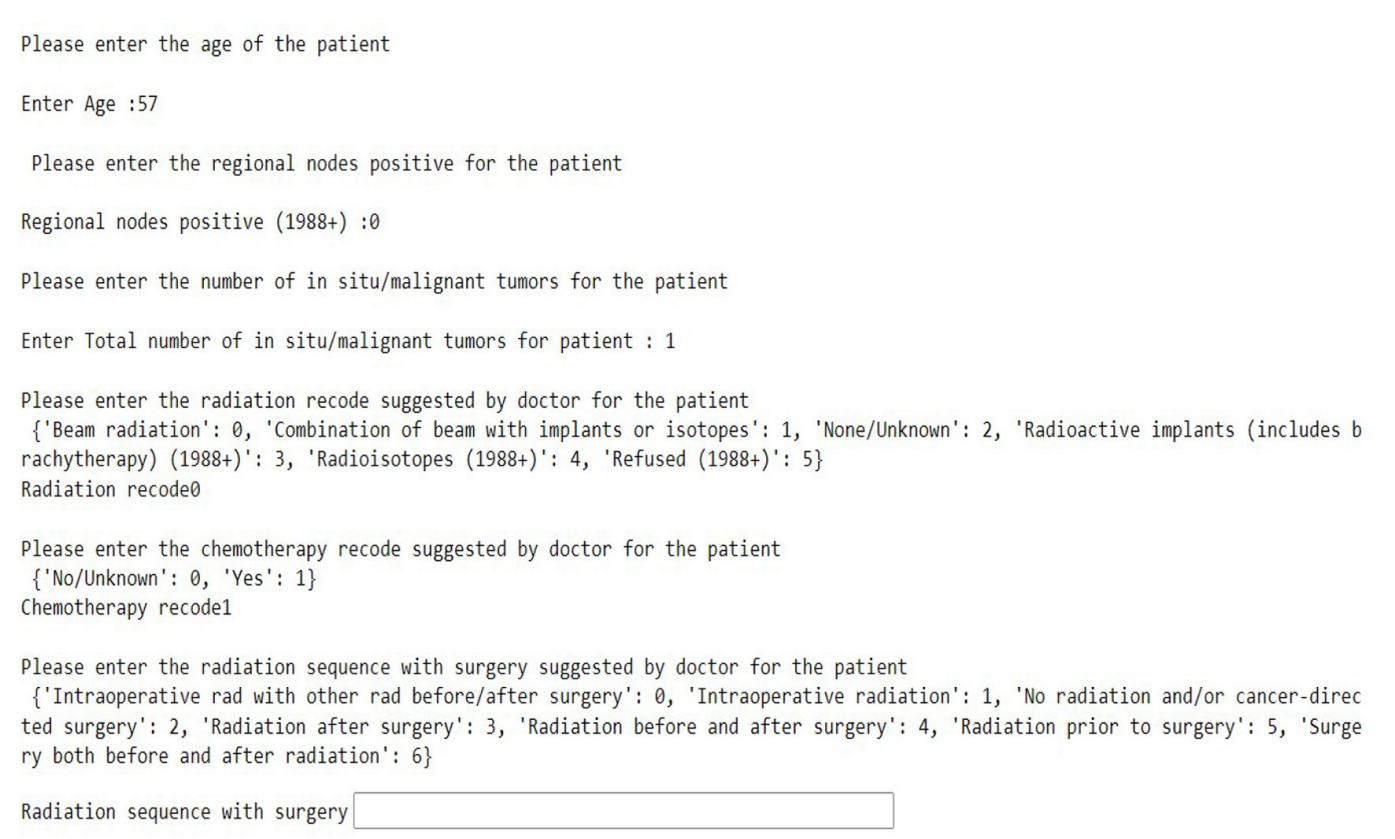
Interactive model.

This model hence was able to predict how different treatments and combinations of various treatment plans can change the prediction of a patient's survival probability. Our model was able to predict what would be the best treatment plan based on the Nearest Neighbors. An interactive model we built is shown in Figure 10, which shows how the doctors can enter patient details to help him/her look at the previous nearest 25 patients' data.

One of the limitations of our study has been the lack of biomarkers and genetics data, which might have provided better results. Still, this study can serve as the foundation for future works where features like genetics and biomarkers could be taken into account with patient understanding accounted for.

## 6. Conclusion

This article proposed a methodology for developing treatment plans and explaining them to patients using machine learning. We illustrated the application of this methodology, focusing on the treatment of breast cancer. A distinguishing feature of our approach is that the user is the patient, and this imposes some restrictions on the type and form of the proposed solutions. For this, a combination of logistic regression and k-nearest neighbors is used. Logistic Regression is initially used to compute survival probabilities and explain the importance of features. We then find *k*-Nearest Neighbors and use them to explain the choice of treatment plans based on similar patients. We believe that using KNN allows the physician to justify his/her choice of treatment and makes it possible for the patient to understand potential risks and outcomes. Our future work will test this approach in real-world conditions to help expand and improve this methodology with further methods of explaining the results and treatment options.

## Data availability statement

Publicly available datasets were analyzed in this study. This data can be found at: https://github.com/snehasi2703/BreastCancerSurvivalDataset.

## Author contributions

All authors listed have made a substantial, direct, and intellectual contribution to the work and approved it for publication.

## References

[B1] Ak M. F. (2020). A comparative analysis of breast cancer detection and diagnosis using data visualization and machine learning applications. Healthcare 8, 111. 10.3390/healthcare8020111 32357391 PMC7349542

[B2] Alaa A. M. Gurdasani D. Harris A. L. Rashbass J. van der Schaar M. (2021). Machine learning to guide the use of adjuvant therapies for breast cancer. Nat. Mach. Intell. 3, 716–726. 10.1038/s42256-021-00353-8

[B3] Bagui S.S B Pal K. Pal N. (2003). Breast cancer detection using rank nearest neighbor classification rules. Pattern Recognit. 36, 25–34. 10.1016/S0031-3203(02)00044-4

[B4] Bishop C. (2016). Pattern Recognition and Machine Learning. New York, NY: Springer.

[B5] Boeri C. Chiappa C. Galli F. Berardinis V. Bardelli L. Carcano G.. (2020). Machine learning techniques in breast cancer prognosis prediction: a primary evaluation. Cancer Med. 9, 3234–3243. 10.1002/cam4.2811 32154669 PMC7196042

[B6] CDC (2022). Centers for disease control and prevention: Breast cancer. Available online at: https://www.cdc.gov/cancer/breast/basic_info/index.htm/ (accessed December 7, 2022).

[B7] Cover T. Hart P. (1967). Nearest neighbor pattern classification. IEEE Trans. Inf. Theory 13, 21–27. 10.1109/TIT.1967.1053964

[B8] Goldberg S. Pinsky E. (2022). “Building a meta-agent for human-machine dialogue in machine learning systems,” in Advances in Information and Communication, FICC 2022, ed A. Kohei (New York, NY: Springer), 474–487.

[B9] Hastle T. (2018). Elements of Statistical Learning. New York, NY: Pearson.

[B10] Medjahed S. Saadi T. Benyettou A. (2013). Breast cancer diagnosis by using k-nearest neighbor with different distances and classification rules. Int. J. Comput. Appl. 61, 1–5.

[B11] Rajbharath R. Sankari L. K. S. (2017). “Predicting breast cancer using random forest and logistic regression,” in International Journal Of Engineering Science and Computing IJESC, Vol. 7.

[B12] Reddy J. Lindsay W. Berlind C. Smith B. (2018). Applying a machine learning approach to predict acute toxicities during radiation for breast cancer patients. Int. J. Radiat. Oncol. Biol. Phys. 102, 559. 10.1016/j.ijrobp.2018.06.167

[B13] Sarkar M. Leong T. (2000). “Application of k-nearest neighbors algorithm on breast cancer diagnosis problem,” in AMIA Annual Proceedings, 759–763. 11079986 PMC2243774

[B14] SEER (2022). National Cancer Institute: Surveillance, Epidemiology, and Results Program: Variable and recode definitions. Available online at: https://seer.cancer.gov/analysis/ (accessed December 7, 2022).

[B15] Song D. Man X. Li Q. Wang H. Du Y. (2021). A decision-making supporting prediction method for breast cancer neoadjuvant chemotherapy. Front. Oncol. 10, 592556. 10.3389/fonc.2020.592556 33469514 PMC7813988

[B16] Sugimoto M. Hikichi S. Takada M. Toi M. (2021). “Machine learning techniques for breast cancer diagnosis and treatment: a narrative review,” in Annals of Breast Surgery Vol. 7, 1–13.35771379

[B17] van de Sande D. Sharabiani M. Bluemink H. Kneepkens E. Bakx N. Hagelaar E.. (2021). Artificial intelligence based treatment planning of radiotherapy for locally advanced breast cancer. Phys. Imaging Radiat. Oncol. 20, 111–116. 10.1016/j.phro.2021.11.007 34917779 PMC8645926

[B18] World health organization (2022). World Health Organization: International Agency for Research in Cancer. Available online at: https://www.iarc.who.int/cancer-topics/ (accessed December 7, 2022).

